# The impact of a short-period head-down tilt on executive function in younger adults

**DOI:** 10.1038/s41598-022-25123-3

**Published:** 2022-12-03

**Authors:** Said Mekari, René J. L. Murphy, Andrew R. S. MacKinnon, Quinn Hollohan, Samantha C. Macdougall, Molly K. Courish, Derek S. Kimmerly, Heather F. Neyedli

**Affiliations:** 1 Department of Family Medicine, Université de Sherbrooke, Sherbrooke, QC Canada; 2 Centre de formation médicale du Nouveau-Brunswick, Pavillon J.-Raymond Frenette, 18 Rue Antonine Maillet, Moncton, NB E1A 3E9 Canada; 3 School of Kinesiology, Acadia University, Wolfville, NS Canada; 4 Division of Kinesiology, Faculty of Health, School of Health and Human Performance, Dalhousie University, Halifax, NS Canada

**Keywords:** Neuroscience, Physiology

## Abstract

Microgravity has been shown to be a significant stressor on the cardiovascular system and the brain due to the redistribution of fluids that occurs in the absence of gravitational force, but there is scarce literature surrounding the effects of microgravity on cerebral hemodynamics and cognition. Understanding the early effects that simulated gravity has on cognitive function is essential for developing proper physical and cognitive countermeasures to assure safe and effective cognitive/decisions making while astronauts prepare for the initial launch or when they arrive in a microgravity environment. Therefore, this study aims to determine how an acute simulation of microgravity would alter cerebral oxygenation and executive functions. Sixty-five young healthy participants (22 ± 6 years, 21 females) completed a thirty (30) minute horizontal (0^0^ tilt) followed by a 90-min − 6° head-down-tilt (HDT) protocol. Cerebral oxygenation in the prefrontal cortex was monitored throughout the testing session using near-infrared spectroscopy. Cognition was also measured using a computerized Stroop Task. Our results demonstrate that cerebral oxygenation was higher during HDT compared to the horizontal supine position (9.11 ± 1.3 vs. 7.51 ± 1.8, p = 0.02). For the cognitive results, the non-executive performance of the Stroop task remained stable during HDT (652.46 ± 19.3 vs. 632.49 ± 14.5, p = 0.09). However, reaction time during the executive task performance was improved after the HDT (1058 ± 195–950 ± 158 ms, p < 0.01). Our results suggest that an acute bout of simulated microgravity can enhance executive functioning.

## Introduction

For decades, physiological responses to microgravity have been heavily studied due to the significant stress this environment places on the body. Humans have evolved to function in the upright posture with gravitational force influencing numerous physiological systems through this constant downward pulling force^1^. However, in microgravity this downward pulling force is lost, causing a multitude of physiological challenges including a redistribution of fluids from the lower extremities to the upper portion of the body^1,2^. Because there is a dramatic fluid shift from the lower body to the upper body during true and simulated microgravity, researchers hypothesized that this phenomenon would affect the amount of blood and oxygen reaching the brain^3^ and therefore cognitive functions. Although the relationship on earth has been established between cerebral oxygenation and cognition^4^, this relationship during microgravity remains relatively understudied. Acute adaptations and effects of microgravity are necessary to understand in order to provide immediate and long-term countermeasures on board space missions to keep cognitive performance elevated for as long as possible. Head down tilt (HDT) is an experimental model that has been used to simulate the effects of microgravity to study multiple physiological systems. This model has become of interest as a terrestrial-based analog for studying simulated microgravity and testing potential countermeasures to microgravity exposures^5,6^. Bed rest lasting from a few days to a few months and longer could provide a unique setup to investigate the efficacy of treatment and interventions to mitigate adverse effects of microgravity on cognition^7^.

There is conflicting evidence from previous HDT research on cognition. A 1-h simulation of head-down-tilt at − 6° was not a significant enough stressor to disrupt mathematical skills and was also not significant enough to impair performance when working through a variety of critical thinking and reasoning tasks^8^. Similarly, fine motor skills, emotional recognition, and reasoning were slightly impaired after 26-h of head down rest, but other cognitive measures such as critical thinking, reaction time, and substitution were seen to slightly improve^9^. In contrast to these findings, found that executive functioning and reaction time were significantly improved following acute bouts of microgravitational exposure, as produced by parabolic flight^10^. Because the methodology of the study revolved around parabolic flight which is a significant stressor to the vestibular system, hormonal influences such as epinephrine and norepinephrine also led to changes in cognitive performance^10^. Additionally, cognitive and perceptual motor performance did not differ between resting, supine, and head-down-tilt (- 6^0^ HDT) positions, but short-term memory was momentarily enhanced during the HDT position^11^. The lack of consistent findings may be due to the different methods of simulating microgravity and the different cognitive functions tested and possible mechanisms for the effects of microgravity remain unknown.

Due to the microgravity-induced headward redistribution of fluids, an emerging avenue of research has been explored to determine if cerebral hemodynamics are also altered. Right middle cerebral blood flow velocity was elevated during the first 3–6 h of − 6^0^ HDT^12^. Furthermore, oxyhemoglobin concentrations have been reported to increase during − 6^0^ HDT^13^. It has also been reported that during the microgravity stages of the parabolic flight, concentrations of oxyhemoglobin were up to three times higher than baseline values and that concentrations of cerebral oxyhemoglobin increased with time during the microgravity stage and remained elevated during the first few seconds once the microgravity stage was over^3^.

Cerebral oxygenation can directly impact cognitive performance^4^. Given that a greater amount of blood reaches the brain during acute microgravity exposure^2^ leading to an increase in cerebral oxygenation^3^, there may be a corresponding enhancement to cognitive performance. While studies have examined the role of microgravity on cognition and cerebral oxygenation separately, to our knowledge, this would be the first study to examine the simultaneous effects of cerebral oxygenation on cognitive performance during microgravity simulation. It was hypothesized that compared to the supine posture: (1) a short duration − 6˚ HDT session will increase cerebral oxygenation in the prefrontal cortex, and (2) a short duration − 6˚ HDT session will also improve reaction time during an executive function task.

## Methods

### Participants

A total of 65 participants (21 Females) gave their written informed consent to participate in the study. The convenient sampling method was used to recruit our participants. The participants’ anthropometric and resting physiological characteristics can be found in Table 1. All participants were English speaking, right-handed, healthy, and had normal-to-corrected vision. Participants were excluded from our study if they were smokers or had a history of neurological or psychiatric disorders, color blindness, surgery with general anesthesia in the past 6 months, involuntary tremors, epilepsy, or drug/alcohol problems. All criteria were assessed during a telephone screening (using the Physical Activity Readiness Questionnaire (PAR-Q+)) and the first meeting at the research center (methodology based on^4^). The protocol was reviewed and approved by the Institutional Research Ethics Board in the Health Sciences of Acadia University (REB 18–28) and this research was conducted in accordance with recognized ethical standards and national/international laws.

**Table 1:** Participants’ anthropometric and physiological characteristics.

Variables	Mean ± SD
Participants	44 males, 21 females
Age (years)	22 ± 6
Weight (kg)	75.2 ± 11.4
Height (cm)	170.6 ± 8.6
BMI (kg/m^2^)	25 ± 3

*Kg* kilograms, *cm* centimeters, *m* meters, *BMI* body mass index.

### Study design

**Figure 1:**
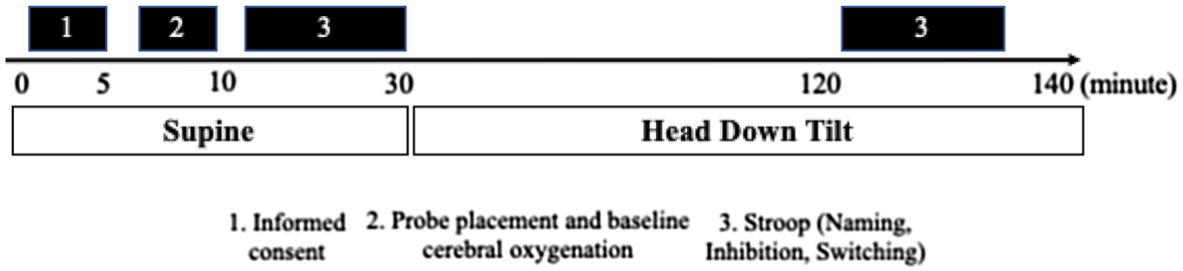
Timeline of procedures during the HDT session.

**Figure 2:**
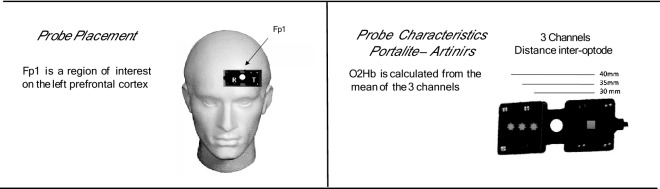
Graphical representation of probe placement, and probe characteristics.

Participants completed one 2.5-h testing session. Upon arrival to the lab, participants signed the consent form and completed questionnaires. A visual representation of the timeline for testing procedures can be found in Fig. 1. Participants completed a 30 min supine baseline condition (0^0^ tilt) followed by a 90 min − 6° head-down-tilt (HDT) protocol. This sequence of testing (supine followed by HDT) was based on previous work in the literature^14^. A computerized Modified Stroop Color Test (see details below) was implemented at the end of the supine and HDT periods. All participants completed their cognitive testing between 10 am and 2 pm. Participants were asked to refrain from moderate (3–6 Metabolic Equivalent (METs)) to vigorous (> 6 METs) exercise 24 h prior to their testing and were also asked to refrain from drinking caffeine (2 h prior to the test) or smoking (2 h prior to the test) and to consume a normal breakfast on the morning of the test. No effort was made to control the nutrition of the participants. Cerebral oxygenation was measured via functional near-infrared spectroscopy (fNIRS) (PortaLite, Artinis Medical Systems, Netherlands) throughout the whole session (see Fig. 2).

### Microgravity simulation protocol

**Figure 3:**
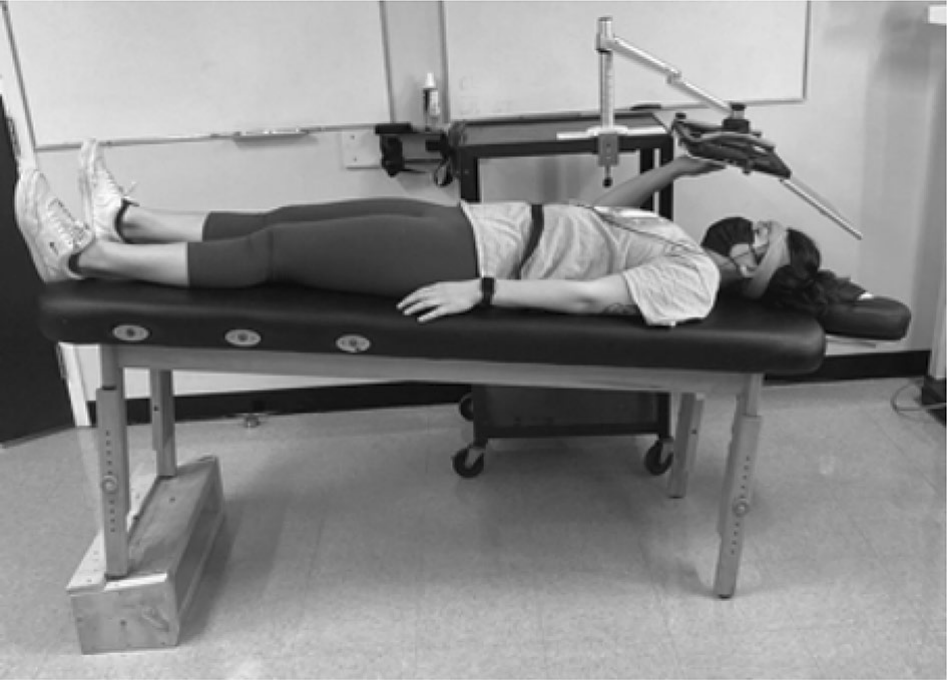
A visual representation of the head-down tilt protocol set-up.

The microgravity simulation involved having subjects lay on a modified tilt table, which was propped up at the foot end to achieve the − 6° of head-down tilt. While there are many angles that researchers will use to see various physiological responses, a − 6° tilt has shown to be the best posture to simulate this microgravity environment^15^. The tilt table used was a cushioned, comfortable therapy table and participants did not have to get up when going from the supine position to the HDT position. The laptop for the computerized Stroop task was attached to a moveable monitor arm that allowed the participants to place the computer in the optimal location to complete the cognition task with their right hand (see Fig. 3).When the participants were not performing the computerized Stroop task, the monitor was moved away from the tilt table. The duration of the tilt was based on previous studies using a − 6° HDT model who showed a decrease of cardiovascular function around the 120 min mark^15–18^. Tomaselli, Kenney, Frey & Hoffler^16^, found that at the acute exposure of a 1-h tilt, cardiac output decreased. These researchers also found that between the minutes of 30–60, it was characterized to be the most significant drop in this variable^16^. Furthermore, Arbeille et al.^19^showed that cardiovascular hemodynamics during a − 6 HDT are significantly different when compared to a supine baseline. Since the objective of the study was to try and understand the role of fluid redistribution on cerebral oxygenation and cognition during a HDT, it was justified for the protocol to always have Supine followed by HDT^19^.

### Computerized stroop task

The computerized Stroop task was based on the Modified Stroop Color Test. This test included three conditions. In the first condition (Naming), the participant read one of four possible color words appearing on the screen (“RED”, “BLUE”, “YELLOW” or “GREEN”). These words were presented in the same colour as their meaning. The colour words were mapped to the corresponding letters on a keyboard: “RED” = “u”, “BLUE” = “i”, “YELLOW” = “o” and “GREEN” = “p”, which participants used to give their answers with their right hand (index finger, middle finger, ring finger and little finger, respectively)^20,21^.The mapping remained the same throughout the task. The second block consisted of a classic Interference task (Inhibition), which required naming the colour of a colour-word, the meaning of the word being incongruent with the font colour (e.g. the word “BLUE” presented in green font). In these first two blocks, a fixation cross appeared for 500 ms, followed by the colour word for 3000 ms. The third block (Switching) consisted of a Switching task, which was identical to the Interference task, except that a square appeared instead of the fixation cross for 25% of the trials, when participants were asked to read the colour-word, instead of naming the font colour. These reading trials appeared randomly throughout the block. Each of the three blocks contained 60 trials and the screen was blank between the trials. Before each condition, participants completed practice trials; 12 for the Congruent condition, 12 for the Inhibition condition, and 20 for the Switching condition. During practice and experimental trials, a visual feedback (“Error”) was given for incorrect responses only. Reaction times and errors were recorded. Reaction times for each trial (60 trials per condition) were averaged for each of the Stroop Test conditions.

### Cerebral oxygenation

A multi-channel continuous wave near infrared spectroscopy (NIRS) system (PortaLite, Artinis Medical Systems, Netherlands) measured relative changes in oxygenated hemoglobin (O_2_Hb) concentration in the brain, The small, non-invasive probe was placed on the participant’s left forehead on the Fp1 prefrontal cortex region, using the 10/20 positioning system. A 2-wavelength continuous measurement system was used in accordance with the absorption characteristics of light, with standard wavelengths of 760 and 850 nm. Optode distances between the receiver and transmitters were 30, 35, and 40 mm. The absorption of which was measured, and concentration changes in O_2_Hb calculated using the difference in absorbance based on the Beer-Lambert law. Because continuous-wave technology does not measure optical path lengths^22^, only changes in concentration of O_2_Hb relative to baseline (i.e. last minute of a 30-min supine period prior to the Stroop Task) could be inferred assuming both a path length factor and partial volume.

The NIRS device was tightly secured with a bandage wrapped around the participant’s head to reduce the amount of interference from device movement or background light. Continuous NIRS measurements were recorded throughout the entire testing session with the average relative concentration of O_2_Hb recorded for each participant. “Events” were manually inserted into the test’s time frame to indicate a change in condition (e.g. “begin Stroop”), to ensure that separation between test conditions and interventions were specified for proper data analysis.

NIRS data were acquired at 10 Hz and filtered with a Savitzky- Golay smoothing algorithm before analysis. All data analyses were completed in the Oxysoft analysis software (Artinis, Netherlands). Data was averaged over every task component (supine, Stroop pre, HDT, Stroop post), and normalized to express the magnitudes of change from the baseline period. The 30-min baseline periods occurred immediately before each Stroop Task assessment; participants sat quietly and were asked to close their eyes and eliminate extraneous thoughts to establish a 60 s baseline of NIRS data (i.e. last 60 s of each 30 min rest period). Cerebral Oxygenation data is presented in arbitrary units (a.u.).

### Statistical analysis

Participants were excluded from analysis if there was an error in recording cerebral oxygenation or response time. Six participants were removed from analysis leaving 59 complete data sets. All statistical analyses were done on SPSS v.25 for Mac. The significance level was set at *p* < 0.05 for all analyses. Standard statistical methods were used for the calculation of means and standard deviations. The normal Gaussian distribution of the data was verified by the Shapiro–Wilk test, and homoscedasticity was verified by a modified Levene’s Test. The compound symmetry, or sphericity, was checked by Mauchly’s test. When the assumption of sphericity was not met, the degree of freedom of F-ratios were adjusted according to the Greenhouse–Geisser procedure when the epsilon correction factor was < 0.75, or according to the Huynh–Feldt procedure, when the epsilon correction factor was > 0.75. Reaction times and cerebral oxygenation were submitted to a 3 (Stroop Task: Naming, Inhibition, Switching) × 2 (Timepoint: Supine vs. HDT) repeated measures ANOVA. Bonferroni corrected paired *t *tests were used for post hoc analysis.

## Results

There was a main effect of Stroop Task, F(1.57,90.8) = 290, p < 0.001, η^2^ = 0.83, and Timepoint, F(1,58) = 32.4, p < 0.001, η^2^ = 0.36, on response time but these main effects were superseded by the two way interaction, F(1.92, 111.7) = 15.1, p < 0.001, η^2^ = 0.21. For the main effect of Stroop tasks, all means were significantly different from one another (all, *p* < 0.0001) with the Switching task (M = 966 ms; SD = 176.48 ms) having the longest response times followed by the Inhibition (M = 761 ms; SD = 146.9 ms) and Naming tasks (M = 645 ms; SD = 112.04 ms). For the interaction there was only a significant improvement in response time pre- to post-HDT for the Switching task, t(58) = 6.84, p < 0.001, d’ = 0.89, but not for the Inhibition task, t(58) = 1.54, p = 0.128, d’ = 0.20, or Naming task t(58) = 1.76, p = 0.08, d’ = 0.23.

**Figure 4:**
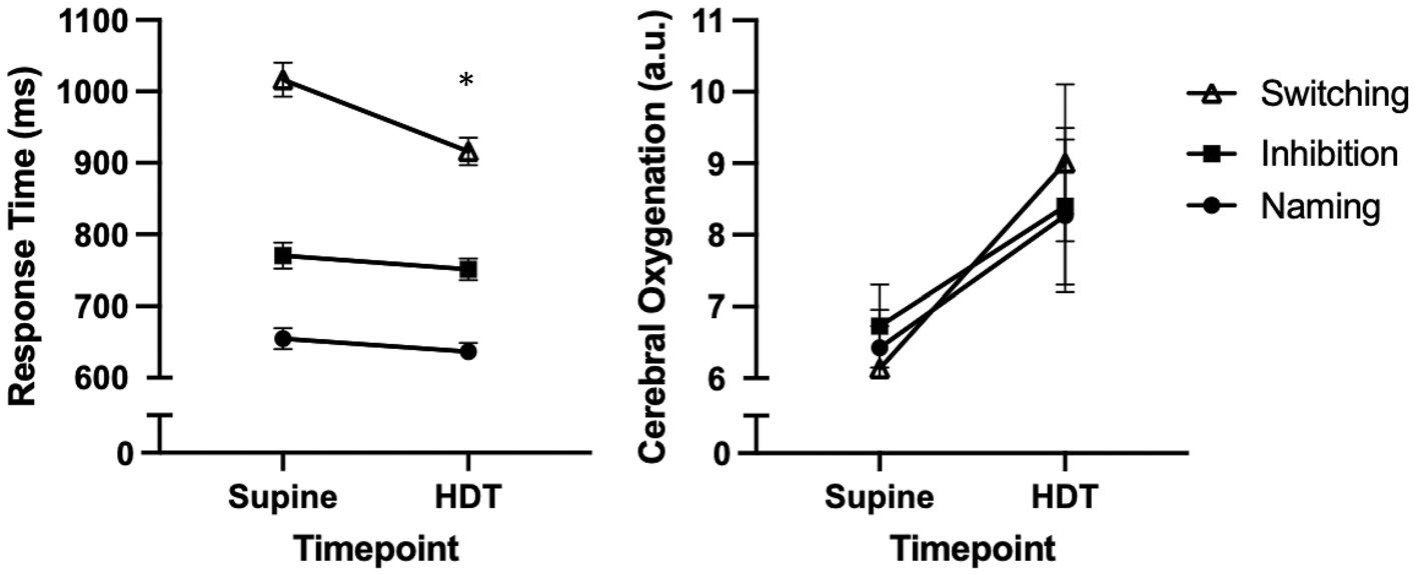
Response time and cerebral oxygenation levels in three Stroop task pre and at end of head down tilt. Error bars are standard error of the mean. *Different from Supine Switching, *p* < 0.05. *a.u* arbitrary units; milliseconds.

For O_2_Hb, there was only a main effect of timepoint, F(1,57) = 6.27, p = 0.015, η^2^ = 0.10, with O_2_Hb increasing pre- to post-HDT in all Stroop Tasks. The main effect of Stroop Task, F(1.68,95.8) = 0.335, p = 0.68, η^2^ = 0.006 and the interaction, F(1.93,110.4) = 2.32, p = 0.105, η^2^ = 0.04, were not significant. Visually, (Fig. 4), there appears to be larger increase in O_2_Hb in the switching task even though the interaction did not reach significance. Paired *t* tests confirmed a Bonferroni-corrected significant difference pre- to post-HDT in the Switching task, t(58) = 3.04, p = 0.003, d’ = 0.40, but not for the Inhibition task, t(58) = 1.99, p = 0.051, d’ = 0.26, or Naming task t(58) = 2.06, p = 0.044, d’ = 0.27.

## Discussion

The purpose of the present study was to determine if an hour and a half of simulated microgravity using a head-down-tilt model would affect cerebral oxygenation and cognitive performance compared to the supine position. Cerebral oxygenation levels increased between the horizontal and HDT conditions (Fig. 3) and that the increase was the largest in the switching condition of the Stroop Task. Furthermore, only the cognitive performance of the executive (Switching) component of the Stroop Task improved during HDT (Fig. 3).

Our first hypothesis was that cerebral oxygenation would be highest following an hour and a half of simulated microgravity by using the head-down-tilt (HDT) technique when compared to values obtained in the supine position, which was partially supported by our results. In the current results, cerebral oxygenation increased during measurement consistently across all points it was measured during the post-HDT Stroop Task indicating that this was a global effect. We also found that the difference in cerebral oxygenation post-HDT appeared to be larger in the switching task even though the interaction did not reach significance. Further analysis revealed significant difference pre- to post-HDT in the Switching task but not for the Inhibition task or the Naming task. Similar results for cerebral oxygenation were reported with previous microgravity studies. These studies confirm that simulated microgravity (i.e. parabolic flight, HDT) increases cerebral oxygenation due to the increased blood flow to the head^3,14^. Previous research has shown that increased neural activation can occur in the brain in the presence of increased cerebral blood and oxygen delivery, such as what occurs during microgravity^2,23^. This increased neural activation can create various cognitive advantages as cells and neurons in the brain receive a greater supply of oxygen necessary for cellular metabolism and optimal functioning. These advantages can include faster decision making, decreased reaction time, and increased resistance to neural fatigue^24^. This increased capacity for cellular functioning might help explain the increased cognitive performance at the end of the HDT in the present study. The increased cerebral oxygenation might have led to increased neural firing frequency resulting in the observed improvement in reaction time. The link between cerebral oxygenation and improvements in cognitive function have been shown before where decision making and reaction time were highest among participants with the greatest levels of cerebral blood delivery^24^; increasingly difficult executive function tasks required increased levels of cerebral oxygenation^4^; and the lowest levels of cognitive performance were correlated with low levels of cerebral blood flow^25^.

Our second hypothesis was that executive function would improve after an hour and a half simulation of microgravity. This hypothesis was also partially supported by our results. We observed an improvement in the switching condition of the Stroop task. Critically, the switching task targets executive function, while the other two tasks are simpler and require less executive function. Our results are similar to executive function improvements shown with microgravity flights where the microgravity portion led to overall decreases in reaction time, and better problem-solving abilities during the microgravitational phase of their flight^10^. A more recent study by Wollseifen and collaborators also reveal that during a parabolic flight, the reaction time in a complex mental task is reduced during weightlessness. They hypothesized that during the microgravity phase of the parabolic flight, an increase in blood volume was responsible for the increase of cortical excitability^10^.

The duration of the intervention is important because of the factors that can influence cognitive performance during sustained microgravity or prolonged bed rest may differ from those of shorter duration microgravity exposures.

It can therefore be concluded that an acute bout of simulated microgravity exposure can momentarily enhance cognition and executive functioning. Based on the findings of the present study and previous research, this improved cognitive performance might be caused by the increased cerebral oxygenation and cerebral blood flow as gravitational force is lost and fluids are redistributed towards the upper portion of the body. However, further research is needed to investigate further mechanisms by which HDT can improve cognition^2,23,25^.

### Limitations of the study

The present study followed many of the accepted methodologies and measurement techniques that currently exist in the literature. However, a few limitations should be noted. First, there were no pre-screening measurements of brain size or total blood volume, which could be responsible for certain variations in cerebral oxygenation. We measured O2Hb in the left prefrontal cortex. The frontal cortex is an area targeted for changes in executive functions^26^. Therefore, our interpretation of the cognitive data is limited to our choice of brain area and observing the contributions of additional areas involved in the Stroop Task might extend the current findings. Compared with the other neuroimaging methods, NIRS has both some important strengths and some notable limitations. On the one hand, NIRS is a noninvasive and relatively low-cost optical technique that has become a widely used instrument for measuring changes in HbO2 particularly during exercise and movement^20^. It can indirectly measure brain blood flow and provides good temporal resolution. On the other hand, NIRS does not have a good spatial resolution and the depth coverage is limited, so most NIRS investigations are limited by skull thickness, scalp flow or adipose tissue thickness^22^. Also, there was no monitoring of cerebral blood flow or cerebral blood flow velocity due to the unavailability of a Doppler Ultrasound device. Thus, there was no way to determine if the variations in cerebral oxygenation were due to cerebral adaptation or varying levels of oxygenated blood reaching the brain.

Secondly, we observed a small statistical improvement in reaction time (100 ms). Although this reaction time might be small, in time critical environments (i.e. astronauts) a reduction of this magnitude could reduce error and increase system efficiency^26^.Finally, there was only an hour and a half period between the participants completing the Stroop tasks, which may give speculation to a possible learning effect. Although we can’t rule out the practice effects, the computerized Stroop task was heavily reviewed by Erickson et al.^27^, and it was found that there are scarce learning effects with this test due to the lack of correlation between practice and reaction time^27^. Furthermore, our data indicates that an increased frequency of the test did not yield significantly better reaction time or accuracy, demonstrating that there is not a substantial learning effect associated with this cognitive task.

### Future areas of research

The present study demonstrated that short-duration simulated microgravity with a − 6°HDT model can significantly increase cerebral oxygenation. This study also found that increased levels of cerebral oxygenation were not statistically associated with increased cognitive performance and executive functioning during simulated microgravity. Future research should examine the effects of longer duration microgravity on cerebral oxygenation and cognition as the present study only used an hour and a half simulation. This study did not counterbalance the supine and HDT conditions. In order to reinforce the methodology and control for possible practice effects, future studies could also look at having participants performing baseline in a sitting position on day 1 and then randomizing supine and HDT in day 2 or 3. Next, the present study only monitored cerebral oxygenation at the prefrontal cortex. Future studies should try and measure cerebral oxygenation throughout the entire brain rather than just one area to obtain a broader map of cerebral hemodynamics and oxygenation allocation during testing. Finally, examining cerebral oxygen metabolism at the cellular level to ascertain if the increases in cerebral oxygenation during microgravity simulation are due to increased stress on the brain during cognition or simply due to the increased amount of blood reaching the brain would be very interesting.

### Conclusions

The present study is valuable in explaining cerebral hemodynamics and patterns of oxygenation and cognition during simulated microgravity as it is essential that cognitive performance remains as high as possible during acute spaceflight. The link between cerebral oxygenation and cognitive performance is still not well-understood, and future areas of research should examine this more closely. Examining these variables further could lead to better cognitive performance during space missions but may also help in explaining certain mechanisms of cognitive disorders such as Alzheimer’s, dementia, and mental deterioration due to aging.

## Data Availability

The datasets used and/or analysed during the current study are available from the corresponding author on reasonable request.

## Abbreviations

ANOVA: Analysis of variance
fNIRS: Functional near-infrared spectroscopy
HDT: Head down tilt
O_2_Hb: Oxygenated hemoglobin
